# American Dentistry in London

**Published:** 1894-05

**Authors:** Frank M. Wilkinson

**Affiliations:** Boston


### AMERICAN DENTISTRY IN LONDON.[1]

BY FRANK M. WILKINSON, D.M.D., BOSTON.

The object of this short paper is to throw light upon a subject of which comparatively little is known in America, and doubtless much chagrin has been experienced by the profession at large on account of the action of the General Medical Council in London in regard to the two dental schools recently disqualified for registration in Great Britain.

Yet when the facts have been clearly set before the profession, it will not be so surprising that such action was taken, but rather that it was so long delayed. American dentistry in England is advertised most extensively and traded upon by most who practise, or pretend to practise, it simply because of the general admission in England of the superiority of it as compared with that practised by other nationalities. The easy credulity of the English public in this matter leads them to be duped by that which is called American, although those who practise under this title are not of that nationality or schooling. The consequence is, that the grossest maltreatment, to speak plainly, is perpetrated, both in supposed-to-be swell private practices as well as in the advertising "limited" companies, who carry on a trade in dentistry rather than a respectable practice. It is well known by the English dentists that this is a great wrong, not only to them, but to the people who are the victims of this avariciousness; they unscrupulously charge the exorbitant fee because it is "American dentistry," while the truth of the matter is, dentists of ability in the States are not guilty of performing operations such as are classed here as American. As a result, the standard of American dentistry in London is anything but what we could wish, and, as seen by most English dentists, is far from being a credit to those who should be representatives of the profession, capable of upholding its high reputation and standard abroad. Why is this? naturally may be asked. The answer is not very hard to find, when it is known that there are several companies in London, with branches throughout England, who claim to be, and advertise as, Americans, and it is certain that many whom they employ are young men from the States. Every dentist who carries on a respectable practice will most emphatically condemn their

[1] Read before Harvard Odontological Society, January 25, 1894.

methods. Some of the work done is not worthy of any American; yet fee enough is charged to command the best obtainable service from the hands of our experts. Their charges are excessive for what is really bad.

With this state of affairs going on for years, it is not surprising that our standard should be looked upon as no improvement upon that of the English. In private practices, there are many at present who for years worked for these firms, and finally established themselves when their time expired for which they were "bound out," for no one can secure a position who will not sign a contract that binds out of London for so many miles and for so many years. Some of these men go on with the same kind of work, and coin money. Even in the aristocratic West End (which one might think would be free from such) they are to be found. Some who never attended college put "Dr." on their door-plate, and "Doctor of Dental Surgery" appears, in so many words, on advertising cards, in defiance of the law restricting them from practising in Great Britain. There are men who charge five hundred dollars (and it has been paid) for a full denture, gold base and rubber attachment, while if a tooth be a trifle off color, it is cut off and a crown put on at large expense. Many of these charlatans place large fillings over putrescent pulps, while the best is being paid for and supposed to be received. There are many working in this way, against whom the more learned and skilful have to compete, the latter in modest and professional manner, while the former class are obtrusive and gain the patronage of the wealthy and the nobility, for the reason that, being Americans, better services are supposed necessarily to follow. What wonder that the standard is not being elevated! But some there are who are faithful and conscientious, and the English dentist is fair enough to acknowledge a good production from the land of the Stars and Stripes.

Graduates of American schools have practically no opportunity of securing good situations in England at the present time. A law has recently been passed by the General Medical Council forbidding dentists, under penalty of having their names removed from the register, to employ unregistered assistants. The only opening for the graduate is to take the examination for the degree of "L. D. S." (Ireland), "sine curriculo," in Dublin. Three Harvard men have passed successfully, and are now registered. Let those who go to practise in London adhere to the principles they have been taught before leaving this country, and there will be a change for the better.

Let all diplomas be disqualified (as the British diplomas are) when graduates are involved with firms of the aforesaid description, and the day will not be far distant when a different state of affairs will be presented, and firms which bring discredit upon the fair name of American dentistry will have little excuse for their existence.

